# Teaching medical students about children with disabilities in a rural setting in a school

**DOI:** 10.1186/1472-6920-7-12

**Published:** 2007-05-15

**Authors:** Peter Jones, Mal Donald

**Affiliations:** 1 University Department of Rural Health, Faculty of Health, University of Newcastle, Tamworth Rural Referral Hospital, Tamworth, NSW, Australia; 2 Bullimbal School for Specific Purposes, NSW Department of Education, Johnson Street, Tamworth, NSW, Australia

## Abstract

**Background:**

To describe and implement a community paediatric placement in a school setting that teaches undergraduate medical students about intellectual disability that provides benefit to the community and is acceptable to both students and teachers.

**Methods:**

Twenty six 4^th ^year undergraduate medical students of the University of Newcastle completed their Paediatric studies based in Tamworth in 2004 & 2005 including an 8 week placement at Bullimbal School for Specific Purposes. The placement involved the students being actively involved in assisting with the delivery of a variety of activities aimed at improving the motor skills of a group of disabled children. De-identified data were obtained from completed evaluation surveys from 75% (21 of 26) of the medical students and from 100% (5 of 5) of the teachers.

**Results:**

All students and teachers found the placement was acceptable and enjoyed the placement and felt that it gave the medical students a greater understanding of children with disabilities. 80% (4 of 5) of the teachers involved in the program did not feel that its implementation added to their workload and all were enthusiastic to continue with the program.

**Conclusion:**

Medical students can be effectively taught and have a valuable clinical experience in a school setting to learn about children with a disability. This educational innovation has provided a mutual benefit for both the medical students and the school children who participated in the program without impacting on the workloads of teachers.

## Background

In Australian Medical Schools there is an increasing recognition that tertiary hospitals are not able to provide for all facets of a comprehensive undergraduate medical education. Modern medical curricula recognize that student directed learning and community orientation are important components of an undergraduate medical education. Recently in Australia there has been a rapid growth in the delivery of medical education in rural settings which has led to several innovations in community based medical education [1]. This paper will describe an innovation in the delivery of the community based components of the Paediatrics course of the Bachelor of Medicine program of the University of Newcastle delivered in a rural setting.

In Australia over the last decade, there have been several specific programs including the Rural Clinical Schools (RCS), University Departments of Rural Health (UDRH) and Rural Undergraduate Support Committee (RUSC) that have increased the amount of undergraduate medical education occurring in rural settings across Australia[2]. By 2008 the integration of these programs will see approximately 20% of all clinical education of undergraduate medical students occurring in rural communities in Australia.

To deliver a community based undergraduate medical curriculum requires engagement with local services and people. Ideally the process of the students being attached to the community should provide a service to the community and in return the students receive an educational experience. The challenge facing the providers of the current Australian Government strategies to promote rural undergraduate medical education is to make the provision of medical education a benefit and not a burden on the local community.

Bullimbal is a government school located in Tamworth, a large regional town in New South Wales with a population of 55,000 for children with a moderate to severe intellectually disability. The School also caters for children with physical disabilities and for children with severe Autism. There are 24 children aged 4 to 19 years that are educated at Bullimbal. Prior to the introduction of this educational pilot Bulimbal had accepted Medical students coming to visit their school. Each year in Tamworth, approximately 8 students would visit for about 1 hour during their paediatric placement.

Although the staff of the school was happy to conduct these "tours" the task was seen as burdensome and the students did not value this opportunity during their paediatric placement. The community aspects of the paediatric placement at that time were largely a series of "visits" to observe various community services but did not involve the students doing anything active.

In 2004 the Newcastle curriculum was changed and Paediatrics and Child Health was taught as a single continuous 8 week block of the fourth year of the Undergraduate Medical Degree. Students were placed in Tamworth in groups of four students. The community placements in Tamworth were reviewed and it was decided to try and make the community placement more active. There is a well documented reduction in the burden of acute infectious diseases in Australian Children and recognition of an increasing burden of developmental, behavioural and mental health problems occurring in children [3]. Recent surveys indicate inadequate training and exposure to these problems even amongst consultant paediatricians [4]. To prepare undergraduate students and give them the core skills and knowledge in these contemporary child health issues requires exposure to child health in settings outside of acute care and tertiary children's hospitals. This educational innovation is one example of how you can expose students to child health issues in the community.

One of the aims of this innovation was to give medical students an understanding of the crucial role that the education system has in caring for children with disabilities. Another aim of the innovation was to try and help medical students gain a greater understanding through a longitudinal exposure to children. A third aim was to design an attachment that was seen as providing a benefit to the children that the medical students worked with and seen as being valuable by the teaching staff of the school. This paper describes an innovation in the medical education program that has enabled a special school to deliver a Motor skills program for their children and the same time allowed several groups of medical students to gain a valuable insight into the care and special needs of children with disabilities.

## Methods

In 2004 and 2005 up to 16 students a year were offered the opportunity to complete the Paediatrics and Child Health course of Newcastle University's Bachelor of Medicine program in Tamworth. Students completed the 8 week placement in groups of 3 or 4. The students were all volunteers. The students based in Tamworth completed a parallel curriculum but were assessed using the same instruments as students based in Newcastle. The attachment was supervised by specialist consultant general paediatricians who worked as staff specialists at Tamworth Rural referral hospital. In the attachment the students gained clinical experience in a 16 bed children's ward and 7 bed neonatal unit with the opportunity to participate in outpatient paediatric sessions. The clinical experience was supported by weekly problem based learning and bedside teaching sessions.

A component of the course delivered in Newcastle included a series of 4 visits to community services that each lasted approximately 1.5 hours. Approval to substitute single visits to several community sites with a number of visits to the one site was gained from the Bachelor of Medicine program committee and the Discipline of Paediatrics and Child Health on the condition that the student experience was evaluated.

The community component of the Paediatric course is compulsory. It is assessed by students attending all rostered community visits. The students based in Tamworth were not given an alternative community visit. They were assessed by attendance. Students and staff that participated in the educational pilot and were asked to complete an anonymous feedback questionaire. Completion of the feedback questionnaire was voluntary. Those that completed the questionnaire gave their consent to having the results used for publication.

The principal of Bullimbal special school designed a placement where up to four students came to the school at the same time for a period of 90 minutes each week. The objective of the community placement was to give the students an insight into the care required for children with special needs in the hope that they would have a greater understanding of the challenges which confront the parents of these special children. This extended attachment was introduced as pilot over 2004 and 2005 to seven groups of fourth year students. The planned schedule of activities which the students undertook included the following:

• In the first week students would be orientated to the school and meet and spend time with the child they were to predominately work with over the ensuing 8 weeks of the placement. Students would also meet staff at the school.

• For the next 7 weeks, students assisted a physiotherapist in helping to transport 5 of the more able bodied children at Bullimbal to the Police Citizen's and Youth Club Gymnasium approximately 1.5 klms away in the school bus.

• The students were then available to help contain the children in the Gymnasium and allow the children to receive an active motor skills program that would have been impossible to deliver as a lone therapist.

• This activity also freed other teaching staff from duties to allow other work at the school to progress.

• Students completed their questionnaire at the end of each attachment and staff completed their questionnaire at the end of 2005.

During the first rotation of students, one of the children at the school had a grand mal epileptic seizure. The medical students administered appropriate first aide to the child and were found to have reacted very appropriately to a stressful emergency situation. There were no other adverse events that occurred during the exercise sessions. Over a 2 year period, the placement permitted a total 42 extra activity sessions to be delivered to this group of children. There were 7 weeks in the two year period of the pilot where the students were completing their attachment during the school holidays and another occasion when the designated day fell on a "pupil-free" day. On average students attended the school 7 times during each attachment for approximately 10.5 hours of time.

## Results

A standard feedback questionnaire was developed that included four questions that used a 5-point Likert Scale. The range was strongly disagree, disagree, neutral, agree and strongly agree. The students were asked if the placement at Bulimbal was a positive experience, if the placement gave the students a greater understanding of children with special needs, if the placement was relevant to Paediatrics and Child Health and if it should be assessed. The staff were asked to give an overall rating for the placement, if the placement gave the students a greater understanding of children with special needs and was a good opportunity to teach the next generation of doctors about children with special needs, if the placement was relevant to Paediatrics and Child Health and if having the students adversely affected their workload. The students and staff were then asked to give their written comments about the placement. Their comments are shown in table 1.

**Table 1:** Written Comments from students and staff who participated in the innovation

**Student Comments**	**Staff Comments**
"I think this placement is really great. Much better being in the one place each week and feeling as though we are involved." "Only problem was not enough time and trouble getting activities organized." "The kids, teachers and staff were appreciative of us helping." "Helping out with gym classes, bowling etc gives you a better understanding of the needs of these children and the resources required to care for them." "Found this placement very valuable. It was great to get to know the kids over the weeks and I found it very rewarding to know their names and have them recognise us every week." "The Kids were great" " Time spent in classrooms prior to sport time didn't seem very well spent (for us to be there – that is)"	"The placement offered the medical students some insight into the life of a disabled person. I feel this would assist them in understanding the difficulties that the carers/parents possibly face." "So far the placement has been a great experience for students and staff. It has allowed us to conduct activities that, due to supervision requirements, would not have been possible without the support of visiting medical students." "That the medical students were able to get to know the children well enough to be able to work confidently with them." "Students learnt and understood the various communication strategies now used with the students who are non-verbal." "The placement will allow the students to remember their interactions with our students so when they are practicing medicine and a family with a child with a disability arrive in their surgery or Emergency Department that they will have a stronger empathy due to their experience in Bullimbal." "Having the extended placement medical students allowed staff at Bullimbal to take students to the Gym and Tenpin Bowling on a ratio of 1:1 which is wonderful therapy for our children." "The medical students completing their placement with us has been a pleasure and most helpful. If this is how our new Doctors are going to be then we are very happy."

21 of 26 students who participated in the attachment completed feedback questionnaires. 10 students agreed and 11 strongly agreed that the placement was a positive experience. All 21 strongly agreed that the placement gave them a better understanding of children with special needs. 2 students were neutral and 19 either agreed or strongly agreed that the placement was important to Paediatrics and Child Health. 4 were neutral and 17 agreed or strongly agreed that completing the placement should contribute towards assessment.

There was five staff that completed questionnaires at the end of 2005. Four staff strongly agreed and 1 staff agreed that the placement was a positive experience. Four staff strongly disagreed that the placement had increased their workload and one was neutral. All strongly agreed that it was an opportunity to teach the next generation of doctors an understanding of people with special needs felt and that the attachment was very relevant to the study of paediatrics and child health.

The evaluation of the placement has been considered positive and the placement has continued to remain an important part of the Paediatrics and Child Health course delivered in Tamworth. The students as part of their evaluation have the opportunity to provide both written and verbal feedback about their attachments.

## Discussion

This paper demonstrates that this type of active community placement is seen as a positive experience by both the students and the teachers involved in its implementation. The opinions of staff were also encouraging because through this attachment the students have been seen as an extra resource for the school and implementing this type of educational program has not been seen as another burden placed on education staff with very demanding professional roles.

A limitation of this study is that it has only looked at a small sample of students participating at just one school and is a description of an educational innovation from the perspectives of the medical students and the teachers of the children at the school. The evaluation of the innovation did not include trying to measure any improvement in the children at the school or if the children enjoyed the activity and relied on the perceptions of the staff and medical students. Such a study was beyond the scope of a quality assurance evaluation of this educational activity. There is research that has documented how clients involved in clinical education can benefit from the experience [5]. To further expand on an approach where medical student education became a measurable benefit to the community would be an exciting future research project. There is a need for Universities in the way they provide education to be both socially accountable and community orientated. This innovation shows a mutual benefit for both the medical students and the staff of the school can accrue from an educational innovation that has not been constrained by the usual boundaries that exist around the hospital based delivery of clinical and academic training for health professional students.

Previously the teaching of Paediatrics at Australian Universities has predominantly taken place in Tertiary Children's Hospitals where paediatricians usually practice as sub-specialists. The advent of the RCS program has demonstrated that students can effectively learn their paediatric clinical skills outside of the tertiary setting and that their paediatric clinical skills may be effectively learnt in a primary care medical setting [6]. This pilot community placement demonstrates how other professionals from outside of health can contribute effectively to the Paediatric education of medical students and may give students a broader understanding of the chronic care issues facing the families of children with disabilities.

Previous studies on this issue have tried to achieve this through looking at students understanding of language [7] or trying to understand the child and parent's perspective of illness[8]. This educational innovation demonstrates that these skills can be learnt outside either a hospital or primary health care setting and may be experienced in an educational community setting. This is consistent with attempts to broaden undergraduate medical student experiences [9].

In Australia there is a current medical workforce shortage [10] and the government is hoping to address this problem by training more doctors. In the next five years there is going to be a doubling in the number of medical students in Australia and there will be a need to think of new ways and places to train these extra undergraduate medical students. This paper has described an innovative partnership with a school as one of the potential new places where students can obtain valuable clinical experience.

## Competing interests

The author(s) declare that they have no competing interests.

## Authors' contributions

PDJ designed the feedback questionnaire and write up of the article. MD designed the activities the students undertook during their placement at the Bulimbal School and reviewed the manuscript prior to submission.

## Pre-publication history

The pre-publication history for this paper can be accessed here:


